# Health-related quality of life in patients accessing medicinal cannabis in Australia: The QUEST initiative results of a 3-month follow-up observational study

**DOI:** 10.1371/journal.pone.0290549

**Published:** 2023-09-06

**Authors:** Margaret-Ann Tait, Daniel S.J. Costa, Rachel Campbell, Richard Norman, Leon N. Warne, Stephan Schug, Claudia Rutherford

**Affiliations:** 1 Faculty of Science, School of Psychology, The University of Sydney, Sydney, New South Wales, Australia; 2 Faculty of Medicine and Health, Sydney Nursing School, The University of Sydney, Sydney, New South Wales, Australia; 3 School of Population Health, Curtin University, Perth, Australia; 4 Little Green Pharma, West Perth, Western Australia, Australia; 5 College of Science, Health, Engineering and Education, Murdoch University, Perth, Western Australia, Australia; 6 School of Pharmacy and Biomedical Sciences, Curtin Health Innovation Research Institute, Curtin University, Perth, Western Australia, Australia; 7 School of Medicine and Pharmacology, University of Western Australia, Perth, Australia; University of Technology Sydney, AUSTRALIA

## Abstract

**Aims:**

Patients with chronic health conditions not responding to conventional treatment can access medicinal cannabis (MC) prescriptions from clinicians in Australia. We aimed to assess overall health-related quality of life (HRQL), pain, fatigue, sleep, anxiety, and depression in a large real-world sample of patients accessing prescribed medicinal cannabis. We hypothesized that all patient-reported outcomes (PROs) would improve from baseline to 3-months.

**Methods:**

The QUEST Initiative is a large prospective multicenter study of patients with any chronic health condition newly prescribed medicinal cannabis between November 2020 and December 2021. Eligible patients were identified by 120 clinicians at medical centers across six Australian states. Consenting participants completed the EuroQol Group EQ-5D-5L health status questionnaire; European Organization for Research & Treatment of Cancer Quality of Life questionnaire (QLQ-C30); Patient-Reported Outcomes Measurement Information System (PROMIS) Short Forms in Fatigue and Sleep Disturbance, and the Depression Anxiety Stress Scale (DASS-21) before starting therapy, at 2-weeks titration, then monthly for 3-months.

**Results:**

Of the 2762 consenting participants, 2327 completed baseline and at least one follow-up questionnaire. Ages ranged between 18–97 years (mean 51y; SD = 15.4), 62.8% were female. The most commonly treated conditions were chronic pain (n = 1598/2327; 68.7%), insomnia (n = 534/2327; 22.9%), generalized anxiety (n = 508/2327; 21.5%), and mixed anxiety and depression (n = 259/2327; 11%). Across the whole cohort both EQ-5D-5L utility scores and QLQ-C30 summary scores showed clinically meaningful improvement in HRQL from baseline to mean follow-up with d = 0.54 (95%CI:0.47 to 0.59) and d = 0.64 (95%CI:0.58 to 0.70) respectively; and clinically meaningful improvement in fatigue (d = 0.54; 95%CI:0.48 to 0.59). There was clinically meaningful reduction of pain for those with chronic pain (d = 0.65; 95%CI:0.57 to 0.72); significant improvements for those with moderate to extremely severe anxiety (*X*^2^ = 383; df = 4; p<0.001) and depression (*X*^2^ = 395; df = 4; p<0.001); and no changes in sleep disturbance.

**Conclusions:**

We observed statistically significant, clinically meaningful improvements in overall HRQL and fatigue over the first 3-months in patients with chronic health conditions accessing prescribed medical cannabis. Anxiety, depression, and pain also improved over time, particularly for those with corresponding health conditions. The study continues to follow-up patients until 12-months to determine whether improvements in PROs are maintained long-term.

**Trail registration:**

**Study registration** - Australian New Zealand Clinical Trials Registry: ACTRN12621000063819. https://www.australianclinicaltrials.gov.au/anzctr/trial/ACTRN12621000063819.

## Introduction

More than 47% of Australians suffer from chronic health conditions [1] and nearly 20% live with persistent chronic pain [2], negatively affecting their Health-Related Quality of Life (HRQL). In 2016, Australian legislation changes allowed patients with health conditions not responding to conventional treatment to access medicinal cannabis (MC) prescribed by clinicians with approval from the Therapeutic Goods Administration (TGA). This decision, driven by patient advocacy groups and community support [3], acknowledged only moderate-quality evidence that MC reduced chronic pain [4], and spasticity in multiple sclerosis [5,6], and low-quality evidence of benefit in chemotherapy-related nausea, vomiting, weight gain in HIV, sleep disorders and Tourette syndrome [7].

Assessing and managing chronic conditions requires consideration of patient-reported outcomes (PROs) [8]. A PRO is any report coming directly from patients about their health status, without interpretation by clinicians or others [9], including symptoms, functioning, and multidimensional constructs such as HRQL. HRQL is defined as: “a multidimensional construct encompassing perceptions of the impacts–positive and negative–of a disease or its treatment on physical, emotional, social, and cognitive functions, as well as somatic discomfort and other symptoms” [10]. PROs are the gold standard for assessing pain [11], and important when assessing chronic conditions where the primary aim is to palliate symptoms [8]. PROs are assessed with PRO measures (PROMs) ‐ validated questionnaires allowing comparisons between groups and over time. PROM-based evidence is encouraged by regulatory bodies internationally [9], and the Australian Commission on Safety and Quality in Health Care recommends using PROMs to drive quality improvement [12].

Real-world data on the effects of MC on HRQL is limited, and patients studied in controlled clinical trials seldom represent the range of chronic health conditions seen in practice [13]. Considering the TGA has approved MC prescription applications for over 200 health conditions [14]. real-world evidence from patients prescribed MC is needed to truly gauge how HRQL changes in practice and inform regulation and policy-making [13,15].

This study is reporting the 3-month interim results of the QUEST initiative (**QU**ality of life **E**valuation **ST**udy), which is assessing patient-reported overall HRQL, pain, fatigue, sleep disturbance, anxiety, and depression for 12-months in a large sample of chronic health patients in Australia prescribed MC. We hypothesized that all PROs would improve from baseline to 3-months in patients accessing MC, and that patients with specific conditions would see improvements in symptoms related to those conditions.

## Methods

### Study population and design

The QUEST initiative is an Australia-wide, multicenter, prospective study of patients with chronic health conditions newly prescribed MC by 120 clinicians between November 2020 and December 2021. Patients were eligible if they: a) were prescribed Little Green Pharma (LGP) MC oil products by a medically registered clinician with TGA approval, b) were ≥ 18 years old, c) were able to read and self-complete online PROMs and study documents in English, and d) had not accessed prescribed MC within the previous 4-weeks. Clinicians completed screening forms via the web-based research data capture system, REDCap [16]. Eligible patients were assigned study ID numbers and emailed generic invitations with Participant Information directly from REDCap. The authors had no access to information that could identify individual participants during or after data collection. To enable future health economic evaluation, clinicians prescribed their study-eligible patients LGP products at a study-standardized price, AUD$150/50ml (GBP £88), representing a 15–38% discount on standard retail prices. The four LGP products prescribed contained phytocannabinoids, delta-9-tetrahydrocannabinol (THC) and cannabidiol (CBD), dissolved in a medium chain triglyceride (MCT) carrier oil in the following ratios: LGP Classic 1:20 (1mg THC and 20mg CBD per ml), LGP Classic 10:10 (10mg THC and 10mg CBD per ml), LGP Classic 20:5 (20mg THC and 5mg CBD per ml), LGP Classic CBD 50 (50mg CBD per ml).

Ethical approval was granted by University of Sydney Human Research Ethics Committee (HREC) Project#:20789 and informed written consent to participate in the study was obtained from all participants. Full details for study design, eligibility, recruitment procedures, PROM administration, and sample size calculations are provided in the study protocol [17].

### Data collection

Clinician-completed screening forms collected basic patient demographics, MC products prescribed, clinical characteristics, and up to two health conditions being treated with MC. Consent, demographics, and patient-reported HRQL, pain, sleep, anxiety, and depression were completed electronically by participants. All PROMs were administered at baseline prior to commencing MC therapy, again after titration (approximately 2-weeks after commencing therapy), then monthly for 3 months. The ‘2-weeks after commencing therapy’ timepoint was chosen because it is when the therapy is expected to be titrated to achieve optimal benefit. Monthly follow-up for 3-months was selected because it aligned with TGA guidance for MC monitoring [18], matched The Centers for Disease Control and Prevention guidelines of 3 months or more frequently for chronic pain management [19], and clinical guidelines of monthly assessments for insomnia [20]. REDCap automatically emailed reminders to participants for follow-up assessments. PROMs were made available to participants to complete within 7-days of the assessment timepoint, after which, the timepoint was recorded as a missed assessment. A detailed description of each PROM administered in in Table 1.

**Table 1:** Outcomes assessed and characteristics, scoring, and details of use for patient-reported outcome measures (PROMs) administered to QUEST participants.

Outcome PROM		Number of items	Domains	Rating	Recall period	Scoring	Details
**HRQL**							
	**EQ-5D-5L** Health status questionnaire developed by the EuroQoL Group [21].	5	Mobility, self-care, usual activities, pain/discomfort, and anxiety/depression.	Items rated 1 (no problem) to 5 (extreme problem or inability)	‘today’	EQ-5D responses were transformed using Australian population utility weights [22] and combined to produce a health index ranging from 0 (death) to 1 (perfect health). Negative values reflect a perceived health state worse than being dead. Higher scores indicate better HRQL.	EQ-5D has been used in published cannabis studies of HRQL in people with neuropathic pain [23,24], and irritable bowel disease [25].
	**QLQ-C30** The European Organization for Research & Treatment of Cancer (EORTC) core quality of life questionnaire (QLQ-C30) [26].	30	Functioning (physical, role, emotional, cognitive and social), global health status quality of life, and symptoms (fatigue, nausea/vomiting, pain, dyspnea, insomnia, appetite loss, constipation, diarrhea) and financial impact.	Items rated 1 (not at all) to 4 (very much), except global health items rated 1 (very poor) to 7 (excellent)	past week	A QLQ-C30 summary score [27] was generated from 27 of the 30 items, excluding the global health and financial impact items, to produce a score between 0 and 100. Higher scores indicate better HRQL.	QLQ-C30 was designed for assessing patients in cancer clinical trials, however it has also been used in other health conditions [28–32], the Australian general population [33], and medical cannabis studies in people with cancer pain. [34,35]
	**QLQ-C15-PAL**^**†**^ EORTC quality of life questionnaire for use in Palliative care setting [36].	15	Functioning (physical, emotional), global quality of life, and symptoms (fatigue, nausea/vomiting, pain, dyspnea, insomnia, appetite loss, constipation).	Same as QLQ-C30	past week		QLQ-C15-PAL has also been used in other palliative care settings [37,38], and chosen because the subscales correspond with, and can be analyzed alongside, the QLQ-C30.
**Pain**							
	**QLQ-C30 Pain subscale** (and QLQ-C15-PAL Pain subscale)	2	Pain: *Have you had pain*? and *Did pain interfere with your daily activities*?	Items rated 1 (not at all) to 4 (very much).	past week	Pain scale produces a score between 0 and 100, with a higher score representing greater pain. The QLQ-C15-PAL and QLQ-C30 pain scales are the same, providing pain scores for all participants.	This pain scale has previously been used in studies of palliative care patients [39], diabetes [40], chronic pain [41], and medical cannabis studies in people with cancer pain.[35]
**Sleep disturbance**							
	**PROMIS Sleep Disturbance 8b** The Patient-Reported Outcomes Measurement Information System (PROMIS) Short Form v1.0 Sleep Disturbance 8b [42].	8	Sleep quality, sleep depth, and restoration.	Items rated 1 (not at all) to 5 (very much so).	past 7 days	PROMIS sleep measure generates a T-score with a mean of 50 and standard deviation of 10 in a reference population of the US general population (US 2000 Census) in combination with a clinical sample [43]. Higher scores reflect greater sleep disturbance.^**‡**^	PROMIS Sleep disturbance has been shown to be valid and sensitive to changes in sleep in women with fibromyalgia, a condition associated with widespread pain, fatigue, and poor sleep quality [44].
**Fatigue**							
	**PROMIS Fatigue 13a** PROMIS Short Form v1.0 Fatigue 13a, also known as Functional Assessment of Chronic Illness Therapy–Fatigue Scale (FACIT-Fatigue) [45].	5	Experience of fatigue, and the impact of fatigue on daily activities.	Items rated 1 (not at all) to 5 (very much so).	past 7 days	PROMIS fatigue measure generates a T-score with a mean of 50 and standard deviation of 10 in a reference population of US general population (US 2000 Census) [43]. Higher scores reflect greater fatigue.^**‡**^	FACIT-Fatigue has been validated in the general population as well as in patients with cancer, anemia, and arthritis [46,47].
	**DASS-21** Depression, Anxiety, Stress Scale -21 [48] is a short version of the DASS-42 [49].	21	Three subscales assessing depression, anxiety, and stress.	Items rated 1 (not at all) to 5 (very much so or most of the time).	past week	For consistent interpretation, DASS-21 scores between 0 and 42 were generated by summing the responses and multiplying by 2 to align with the DASS-42 [50]. Higher scores reflect greater symptom burden	DASS-21 is a validated PROM of depression and anxiety used in routine assessment of in-patients [48], patients with multiple sclerosis [51], pain [52], and in the general population [53].
**Depression**							
	**DASS-21 Depression subscale**	7	Dysphoria, hopelessness, devaluation of life, self-deprecation, lack of interest/involvement, anhedonia, and inertia.	Items rated 0 (not at all) to 3 (very much or most of the time)	past week	DASS-depression scores were categorized into severity levels at each timepoint as follows: 0–9 normal, 10–13 mild, 14–20 moderate, 21–27 severe, 28+ extremely severe [50].	
**Anxiety**							
	**DASS-21 Anxiety subscale**	7	Autonomic arousal, skeletal muscle effects, situational anxiety, and subjective experience of anxious affect.	Items rated 0 (not at all) to 3 (very much or most of the time)	past week	DASS-anxiety scores were categorized into severity levels at each timepoint as follows: 0–7 normal, 8–9 mild, 10–14 moderate, 15–19 severe, 20+ extremely severe [50].	

DASS Depression, Anxiety, Stress Scale; HRQL health-related quality of life; PROM patient-reported outcome measure.

^†^To reduce burden, palliative care patients with advanced, symptomatic, incurable conditions, only completed two PROMs at each timepoint (QLQ-C15-PAL and EQ-5D-5L). Non-palliative care participants completed all PROMs at each timepoint (except QLQ-C15-PAL).

^‡^The HealthMeasures Scoring Service recommended for PROMIS instruments was used to calculate T-scores because it uses item level calibrations more accurately than manually transforming total raw scores [54].

### PROMs

We aimed to assess PROs using validated PROMs as described in Table 1. PROMs used to assess HRQL were designed to cover all dimensions of HRQL as defined in the introduction.

### Statistical analyses

#### Statistical significance

For each PRO, participants with a score at baseline and at least one follow-up assessment were analyzed. All PROMs were scored according to standard scoring algorithms provided by the PROM developers.

PROMIS measures generate T-scores with a mean of 50 and standard deviation of 10 in a reference population of the US general population (US 2000 Census) in combination with a clinical sample [43]. The HealthMeasures Scoring Service recommended for PROMIS instruments was used to calculate T-scores because it uses item level calibrations more accurately than manually transforming total raw scores [54]. Statistical analyses were carried out using the IBM SPSS Statistics 28.0 program.

Means, standard deviations (SD), and standardized mean-difference effect sizes with 95% confidence intervals were calculated for each assessment timepoint. Differences in baseline patient characteristics were explored using linear regression on QLQ-C30 scores, and where significant, adjusted for in the longitudinal analyses. Linear mixed models were used to examine change over time in PRO scores, with time included as a random factor. To adjust for possible Type I error inflation due to the analysis of multiple dependent variables, we used the Hochberg adjustment [55]. The model adjusted for PRO levels at baseline and potential confounders, such as cannabis use within previous 12-months and sex, with duration of pain and age modelled as fixed factor covariates. Analyses compared mean scores at baseline with mean scores at each follow-up timepoint and the mean of post-intervention scores, and analyzed trends over time and interactions between groups. In the linear mixed models, we probed change over time in two ways: (1) by analyzing contrasts representing linear and quadratic trends, to determine whether there was constant change over time (linear only) or change at a changing rate (linear + quadratic); (2) by analyzing a contrast comparing baseline scores to the mean of post-intervention scores. Additionally, change in DASS-anxiety and DASS-depression severity categories from baseline to follow-up were analyzed using Pearson Chi-squared.

As per the study protocol [17], a sample size of 2142 was powered to detect the smallest effect size threshold (Cohen’s d = 0.1) of difference in QLQ-C30 insomnia domain [56].

#### Clinically meaningful change

This study, and others with large sample sizes, may detect statistically significant changes that are not large enough to be clinically meaningful or important in practice. A minimally clinically important difference (MCID) is “the smallest difference which patients perceive as beneficial and which would mandate, in the absence of troublesome side effects and excessive cost, a change in the patient’s management” [57]. The EQ-5D-5L index score MCID in general populations falls between 0.037 and 0.069 [58]. While there are recommended QLQ-C30 subscale MCIDs [56], currently there are no published QLQ-C30 Summary Score MCIDs. The recommended threshold for evaluating meaningful within-group change using PROMIS measures generally ranges between 2 and 6 T-score points [59] with PROMIS Group consensus on 3 T-score points. The MCID for DASS-21 depression and anxiety scales is movement from one severity level to another (e.g., from ‘severe’ to ‘moderate’), as well as a change of 5 points [60].

Clinically meaningful differences on PROs over time were interpreted using existing guidelines where available. In the absence of guidelines, the threshold for discriminating HRQL changes for chronic diseases has generally been found to be approximately half of the standard deviation of change score (i.e. Cohen’s d = 0.5) [61]. A threshold of Cohen’s d = 0.5 was used as the MCID for the QLQ-C30 Summary Score.

### Patient and public involvement

Patients were involved in this research as participants providing self-rated PROM responses reflecting their personal experiences while prescribed MC. PROMs used were previously developed and validated elsewhere in collaboration with patients and public who provided feedback on relevance and comprehension of questionnaire items. Patient participation was voluntary. Patients and public were not directly involved in developing the research question or study design. A summary of study findings will be emailed to participants and their clinicians.

## Results

**Fig 1:**
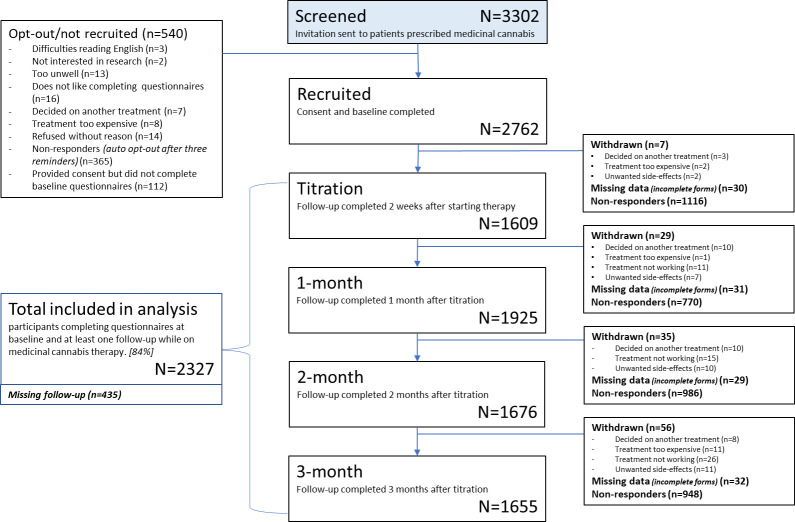
Study recruitment flow.

Of 3302 eligible patients emailed invitations, 2762 provided consent and completed baseline PROMs and demographic information. Of those, 2327 completed at least one follow-up PROM and were included in the analysis (Fig 1). Those who dropped out after completing baseline were generally younger than those who continued. Participants were aged between 18–97 years (mean 51y; SD = 15.4), 62.8% female, and 37.6% University educated. Due to illness, 25.4% were either unemployed, on leave, or on limited work duties (Table 2). S1 Table shows numbers of patients screened and participating from each Australian state and territory, and further demographic information on gender identity and ethnicity.

**Table 2:** Baseline characteristics of 3302 eligible patients invited to QUEST grouped by those screened but not joined, participants completing baseline questionnaires only, and participants included in the analyses (completed baseline plus at least one follow-up).

	Screened - not joined	Completed baseline only	Included in analysis	P value (*Χ*^2^)
Characteristics				
Total *(n = 3302)*	540	435	2327	
**Age (years), *mean (SD)***	51 (17.6)	47 (16.9)	51 (15.4)	**>0.001**
**Sex, *n (%)***				**>0.001**
Male	249 (46.1)	172 (39.5)	863 (37.1)	
Female	291 (53.9)	262 (60.2)	1462 (62.8)	
Indeterminate/Intersex	0	1 (0.2)	2 (0.1)	
**Palliative care, *n (%)***	8 (1.5)	7 (1.6)	27 (1.2)	0.67
**Currently treated for Cancer** (any), ***n (%)***	23 (4.3)	24 (5.5)	98 (4.2)	0.47
**Living arrangements, *n (%)***				0.23
Live alone		84 (19.3)	467 (20.1)	
Live with partner		249 (57.2)	1414 (60.8)	
Live with carer		11 (2.5)	42 (1.8)	
Live with other		88 (20.2)	394 (17.0)	
Live in assisted care home		3 (0.7)	7 (0.3)	
Missing		-	3	
**Marital Status, *n (%)***				**0.02**
Single		116 (26.7)	507 (21.8)	
Married		184 (42.3)	1104 (47.5)	
Separated		24 (5.5)	93 (4.0)	
Divorced		37 (8.5)	251 (10.8)	
Widowed		21 (4.8)	70 (3.0)	
Cohabitating		53 (12.2)	299 (12.9)	
Missing		-	3	
**Work Status, *n (%)***				0.50
Full time		138 (32.2)	658 (28.6)	
Part time		68 (15.9)	361 (15.7)	
At work but limited hours/duties		27 (6.3)	131 (5.7)	
Retired		68 (15.9)	449 (19.5)	
Unemployed due to illness		78 (18.2)	394 (17.2)	
Unemployed NOT due to illness		6 (1.4)	43 (1.9)	
On leave due to illness		10 (2.3)	57 (2.5)	
Home duties		15 (3.5)	99 (4.3)	
Studying only		9 (2.0)	65 (2.8)	
Voluntary work		6 (1.4)	25 (1.1)	
Retraining		4 (0.9)	15 (0.7)	
Missing		6	30	
**Education, *n (%)***				**0.05**
Primary School		8 (1.8)	23 (1.0)	
High School		132 (30.3)	588 (25.3)	
Certificate or Diploma		149 (34.3)	839 (36.1)	
University or higher		146 (33.6)	874 (37.6)	
Missing		-	3	

SD standard deviation; P values in bold are significant.

After titration, median MC daily doses were: LGP Classic 1:20 = 1.0ml (IQR: 0.50, 1.50); LGP Classic 10:10 = 0.75ml (IQR: 0.37, 1.36); LGP Classic 20:5 = 0.57ml (IQR: 0.30, 1.10); and LGP Classic CBD 50 = 1.0ml (IQR: 0.50, 1.63). MC had previously been prescribed for 108/2327 (4.6%) participants (but not within 4-weeks prior to joining the study), and 521/2327 (22.4%) had used cannabis recreationally, or medicinally without a prescription, within 12-months prior to joining.

Half of participants were prescribed MC for more than one health condition (n = 1233/2327; 53%), with the majority treated for chronic pain (n = 1598/2327; 68.7%). Other common conditions included insomnia (n = 534/2327; 22.9%), anxiety (n = 508/2327; 21.5%), and mixed anxiety and depression (n = 259/2327; 11%). S2 Table shows participant health conditions and MC products prescribed at baseline.

### PROs

Analyses, where appropriate, were adjusted for age, sex, duration of pain condition, and exposure to cannabis within the previous 12-months (recreational or medicinal). These patient characteristics were identified as significant covariates using linear regression on baseline QLQ-C30 summary scores, however not all were significant covariates in the longitudinal analyses. For example, exposure to cannabis in previous 12-months rarely predicted changes in PROs over time. Similarly, age was not associated with QLQ-C30 scores over time, however younger age was associated with higher EQ-5D-5L scores. Two participants identified as intersex were excluded from adjusted analyses.

Results in Table 3 summarize the clinical meaningfulness of change scores using Cohen’s d = 0.5 as the MCID threshold for HRQL, pain, sleep, fatigue, depression, and anxiety from baseline to each follow-up timepoint, and baseline to overall post-treatment mean.

**Table 3:** Clinical meaningfulness of change by effect size in patient-reported outcomes from baseline to each timepoint (2 weeks titration, then 1-, 2-, and 3-months post-titration) and mean post-treatment follow-up in patients with any health condition prescribed medical cannabis.

PROM		Mean scores at baseline and each follow-up timepoint												Mean post-treatment overall			
	Baseline	Titration	ES (95% CI)	Baseline	1- Month	ES (95% CI)	Baseline	2- Month	ES (95% CI)	Baseline	3- Month	ES (95% CI)	Baseline	Mean Follow-up	ES (95% CI)	*P* ^c^
**HRQL**^**a**^ **EQ-5D-5L Utility Score**																	
	N	1608	1608	0.46 (0.39, 0.53)	1925	1925		1672	1672		1653	1653		2325	2325		
	Mean (SD)	0.42 (0.29)	0.55 (0.27)		0.42 (0.29)	0.55 (0.28)	0.46 (0.39, 0.52)	0.41 (0.30)	0.58 (0.28)	**0.59** (0.52, 0.65)	0.41 (0.29)	0.58 (0.28)	**0.60** (0.53, 0.67)	0.41 (0.29)	0.57 (0.28)	**0.54** (0.47, 0.59)	<0.001
**QLQ-C30 Summary Score**																	
	N	1574	1574		1900	1900		1648	1648		1629	1629		2297	2297		
	Mean (SD)	60.20 (16.41)	69.74 (16.30)	**0.62** (0.55, 0.69)	59.81 (16.46)	70.26 (16.49)	**0.63** (0.57, 0.70)	59.75 (16.88)	71.02 (17.02)	**0.66** (0.59, 0.73)	59.56 (16.75)	71.16 (17.22)	**0.68** (0.61, 0.75)	59.85 (16.63)	70.54 (16.75)	**0.64** (0.58, 0.70)	<0.001
**Pain** ^**b**^ **QLQ-C30 (or QLQ-C15Pal) Pain subscale**																	
	N	1598	1598		1921	1921		1667	1667		1645	1645		2326	2326		
	Mean (SD)	58.80 (31.71)	47.26 (29.95)	0.37 (0.30, 0.44)	59.42 (31.11)	45.75 (29.29)	0.45 (0.39, 0.52)	59.62 (31.20)	44.70 (29.92)	0.49 (0.42, 0.56)	59.64 (31.10)	43.99 (29.61)	**0.52** (0.45, 0.58)	58.86 (31.34)	45.42 (29.69)	0.45 (0.38, 0.50)	<0.001
**Sleep** ^**b**^ **PROMIS Sleep Disturbance 8b T-scores**																	
	N	1568	1568		1894	1894		1635	1635		1619	1619		2299	2299		
	Mean (SD)	51.27 (3.41)	51.49 (3.39)	-0.06 (-0.13, 0.01)	51.30 (3.42)	51.33 (3.53)	-0.01 (-0.07, 0.06)	51.29 (3.51)	51.24 (3.56)	0.01 (-0.05, 0.08)	51.28 (3.48)	51.32 (3.57)	-0.01 (-0.08, 0.06)	51.26 (3.49)	51.37 (3.5)	0.03 (-0.03, 0.09)	0.29
**Fatigue** ^**b**^ **PROMIS Fatigue 13a T-scores**																	
	N	1568	1568		1895	1895		1635	1635		1619	1619		2299	2299		
	Mean (SD)	58.30 (8.17)	54.50 (8.14)	0.47 (0.39, 0.54)	58.32 (8.07)	54.25 (8.30)	**0.50** (0.43, 0.56)	58.51 (8.09)	53.97 (8.36)	**0.55** (0.48, 0.62)	58.34 (8.09)	53.92 (8.55)	**0.53** (0.46, 0.60)	58.37 (8.11)	54.16 (7.60)	**0.54** (0.48, 0.59)	<0.001
**Depression** ^**b**^ **DASS-21 Depression subscale**																	
	N	1570	1570		1895	1895		1639	1639		1624	1624		2299	2299		
	Mean (SD)	15.10 (10.95)	11.15 (10.06)	0.38 (0.31, 0.45)	15.09 (10.86)	10.90 (9.79)	0.41 (0.34, 0.47)	14.96 (10.83)	10.55 (9.83)	0.43 (0.36, 0.50)	14.97 (10.71)	10.34 (9.81)	0.45 (0.38, 0.52)	15.20 (10.90)	10.73 (9.87)	0.44 (0.37, 0.49)	<0.001
**Anxiety** ^**b**^ **DASS-21 Anxiety subscale**																	
	N	1570	1570		1895	1895		1639	1639		1624	1624		2299	2299		
	Mean (SD)	10.26 (8.67)	7.33 (6.86)	0.37 (0.30, 0.44)	10.42 (8.69)	7.28 (6.97)	0.40 (0.33, 0.46)	10.36 (8.76)	6.94 (7.03)	0.43 (0.36, 0.50)	10.57 (8.74)	7.10 (7.18)	0.43 (0.36, 0.50)	10.57 (8.75)	7.17 (7.01)	0.45 (0.37, 0.49)	<0.001

ES: Standardized mean-difference effect size (Cohen’s d), **bold** indicates clinically meaningful change determined by effect size (d≥0.5).

^a^ Higher scores indicate better HRQL.

^b^ Higher scores indicate worse symptoms.

^c^ p-value for mean difference of baseline to mean post-treatment follow-up across all participants (2-tailed T-test).

### Overall HRQL

**Fig 2:**
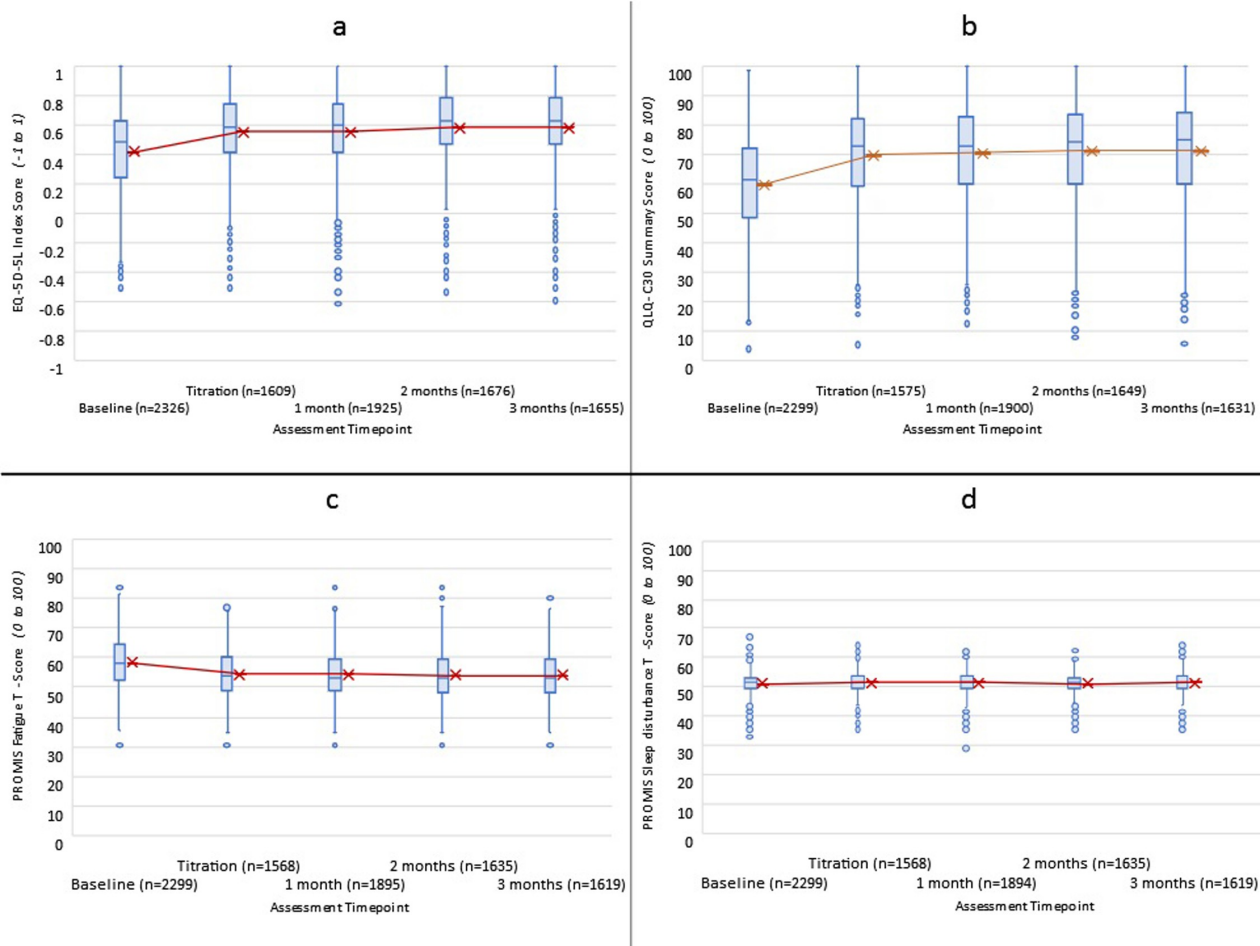
Score distribution (blue) and mean trends (red) from baseline to 3-months following titration for a) EQ-5D-5L Australian weighted Index Scores, b) QLQ-C30 Summary Scores, c) PROMIS-Fat T-scores, and d) PROMIS Sleep disturbance T-scores. Higher scores indicate better quality of life (a & b). Higher scores indicate worse symptom burden (c & d).

EQ-5D-5L index scores (n = 2325) displayed significant linear (t_(9172)_ = 18.45, p<0.001) and quadratic (t_(6697)_ = -16.45, p<0.001) trends over time, showing rapid initial improvement that was maintained over 3-months (Fig 2A). Mean EQ-5D-5L index scores improved by 0.152 (SD = 0.28) from baseline (0.414; SD = 0.29) to mean follow-up (0.566; SD = 0.28), indicating a clinically meaningful improvement (d = 0.54; 95%CI: 0.47 to 0.59). This observed change was greater than the recommended EQ-5D-5L index score MCID for general populations, which falls between 0.037 and 0.069 [45].

QLQ-C30 summary scores (n = 2297) also showed significant linear (t_(8912)_ = 22.79, p<0.001) and quadratic (t_(6513)_ = -23.41, p<0.001) trends of improvement over time(Fig 2B). Mean change in QLQ-C30 summary score of 10.7 (SD = 16.72) from baseline (59.85; SD = 16.63) to mean follow-up (70.54; SD = 16.75) indicated a clinically meaningful improvement (d = 0.64; 95%CI: 0.58 to 0.70).

### Pain

**Fig 3:**
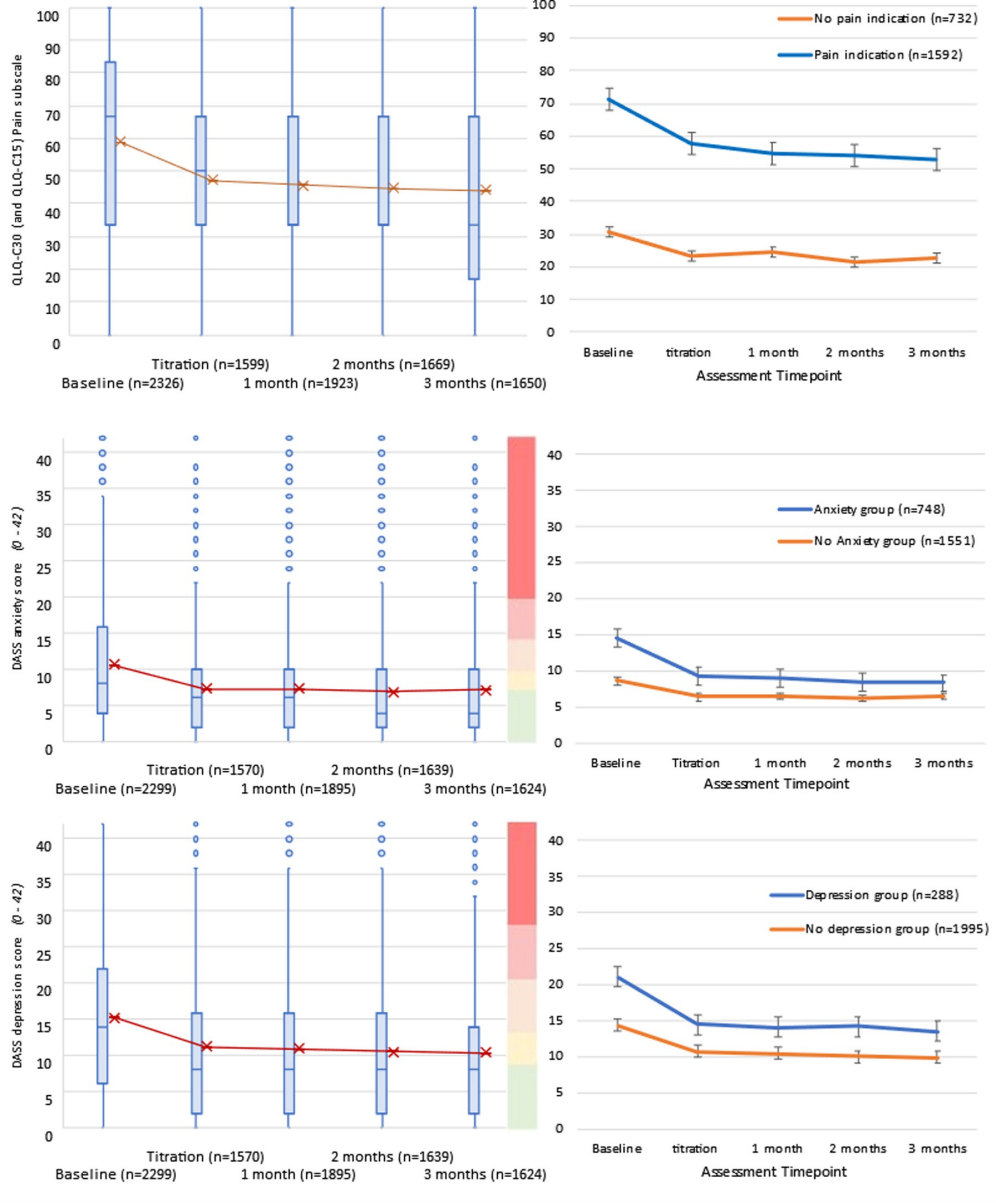
Score distribution and group comparisons of mean scores for Pain, Anxiety, and Depression from baseline to 3-months following titration. Higher scores indicate greater symptom burden. Pain group includes participants with any diagnosed pain condition (listed in Appendix B). Anxiety group includes participants diagnosed with generalized anxiety disorder or mixed depressive and anxiety disorder. Depression group includes participants diagnosed with recurrent depressive disorder, mixed depressive and anxiety disorder, or bipolar disorder.

QLQ-C30 pain scores (calculated from QLQ-C30 or QLQ-C15 PAL) improved from baseline to follow-up with significant linear (t_(9149)_ = -16.73,p<0.001) and quadratic (t_(6693)_ = 14.46,p<0.001) trends of improvement over time(Fig 3). When comparing participants with a chronic pain diagnosis with those not being treated for pain, improvements from baseline to mean follow-up were greater for the pain group (t_(4511)_ = 9.79, p<0.001)(Fig 3).

Looking specifically at the 1592 participants with a chronic pain diagnosis, mean pain scores improved by 16.51 (SD = 26.07) from baseline (71.24; SD = 24.53) to follow-up (54.73; SD = 26.56), indicating clinically meaningful improvement (d = 0.65; 95%CI: 0.57 to 0.72). Following guidelines for interpreting QLQ-C30 subscale change scores, more than 14 points on the pain subscale is regarded as a large improvement [62].

### Sleep

PROMIS Sleep Disturbance, completed by 2299 participants, provided no evidence of statistically significant, or clinically meaningful change in mean Sleep T-scores over time (Fig 2C). Mean baseline scores (T = 51.26; SD = 3.49) deteriorated by 0.11 points at mean follow-up (T = 51.37; SD = 3.5) with an effect size of 0.03(p = 0.29). Similarly, analysis of 534 participants with an insomnia diagnosis (of which 460 (86%) had a secondary diagnosis) did not reveal statistically significant, or clinically meaningful change in mean Sleep T-scores over time and did not differ from patients without insomnia. Mean baseline scores (T = 51.48; SD = 3.40) deteriorated by 0.12 points at mean follow-up (T = 51.60; SD = 3.41) with an effect size of 0.03(p = 0.57).

### Fatigue

PROMIS Fatigue T-scores (n = 2299) displayed significant linear (t_(8995)_ = -16.77,p<0.001) and quadratic (t_(6559)_ = 16.6,p<0.001) trends of improvement over time (Fig 2D). Fatigue improved on average by 4.21 T-scores (SD = 7.73) from baseline (T = 58.37; SD = 8.11) to mean follow-up (T = 54.16; SD = 7.60), indicating clinically meaningful improvement (d = 0.54; 95%CI: 0.48 to 0.59). This improvement was greater than the recommended PROMIS MCID of 3 T-scores.

### Anxiety

Mean DASS-Anxiety scores displayed significant linear (t_(8962)_ = -13.76,p<0.001) and quadratic (t_(6402)_ = 15.29,p<0.001) trends of improvement over time (Fig 3). Mean difference between baseline (10.57; SD = 8.75) and mean follow-up (7.17; SD = 7.01) was 3.4 (SD = 7.49), with d = 0.45(95%CI: 0.37 to 0.49). Although mean scores moved from moderate severity into mild severity range, the difference did not reach the recommended 5-point threshold. Comparing participants with anxiety health conditions with those not treated for anxiety, the improvement in DASS-anxiety scores from baseline to mean follow-up was greater for the anxiety group (t_(8661)_ = 11.71, p<0.001) (Fig 3). After categorizing baseline and average follow-up anxiety scores, comparisons using Chi-square goodness of fit test showed significant movement from more severe anxiety categories towards the normal range (*X*^2^ = 383; df = 4; p<0.001; S1 Fig).

Looking specifically at the 748 participants with anxiety health conditions (i.e. generalized anxiety or mixed depression and anxiety), the mean difference between baseline (14.56; SD = 8.92) and mean follow-up (8.79; SD = 7.59) was 5.77 (SD = 7.96), with d = 0.72(95%CI: 0.59 to 0.80) indicating clinically meaningful improvement. On average, scores shifted from moderate/severe down to mild anxiety with more than 5-point change, meeting the recommended threshold for clinically meaningful improvement.

### Depression

Mean DASS-Depression Scores displayed significant linear (t_(8921)_ = -14.69,p<0.001) and quadratic (t_(6451)_ = 14.33,p<0.001) trends of improvement over time (Fig 3). Mean difference between baseline (15.20; SD = 10.90) and follow-up (10.73; SD = 9.87) was 4.47(SD = 10.14), with d = 0.44(95%CI:0.37 to 0.49). Although scores shifted from moderate severity into mild severity range, the difference did not reach the 5-point threshold for clinically meaningful improvement. Comparing participants with depression health conditions with those not being treated for depression, the change in DASS-depression scores from baseline to mean follow-up was greater for the depression group (t(8601) = 5.30, p<0.001)(Fig 3). After categorizing baseline and mean follow-up depression scores, comparisons using Chi-square goodness of fit test showed significant improvement from more severe categories towards the normal range (*X*^2^ = 395; df = 4; p<0.001; S1 Fig).

Looking at the 288 participants with depression health conditions (i.e. mixed depressive and anxiety, recurrent depressive disorder, and bipolar disorder), mean improvement from baseline (21.04; SD = 11.22) to mean follow-up (14.06; SD = 10.79) was 6.98 (SD = 10.91), with d = 0.64(95%CI: 0.47 to 0.80). On average, respondents shifted from the severe category to moderate depression with more than 5-points difference indicating clinically meaningful improvement.

### Missed assessments

During the 3-month follow-up period, 127 participants formally withdrew from the study. Recorded reasons for withdrawal included changing treatment (n = 31), treatment too expensive (n = 14), treatment not working (n = 52), or unwanted side-effects (n = 30). Follow-up data collected from these participants were included in the analysis up to the time they withdrew, however seven participants withdrew before completing any follow-up assessment (Fig 1).

S2 Fig shows EQ-5D and QLQ-C30 results stratified by those who dropped out or failed to complete follow-up after each timepoint. Participants only completing baseline (n = 435) had poorer HRQL than those who continued on the study (QLQ-C30 Summary score MD = 2.03; SD = 16.8; p = 0.02). Those who only completed baseline and titration showed less improvement in HRQL compared with those who continued. At each follow-up timepoint, between 28% and 40% of participants failed to complete PROMs (Fig 1).

## Discussion

### Principal findings

As hypothesized, this study found overall HRQL improved over 3-months in patients accessing prescribed MC in Australia. Results showed both statistically significant and clinically meaningful improvements in overall HRQL and fatigue for people with chronic health conditions. Similar improvements were found in pain scores for participants with chronic pain; depression scores for patients with depression; and anxiety scores in patients with anxiety. Interestingly, although many patients were prescribed MC for insomnia, no improvements in patient-reported sleep disturbance were observed.

Although HRQL improvement was similar between the EQ-5D-5L index and QLQ-C30 summary scores, differences were observed when looking at responses from participants who dropped out after baseline (S2 Fig), and when adjusting for age. As participant age increased, EQ-5D-5L index scores decreased slightly over time, whereas QLQ-C30 summary scores were not affected. The association between increased age and declining EQ-5D utility scores has previously been reported for the Australian population [63], whereas a previous Australian population study using QLQ-C30 found that increased age was only associated with some QLQ-C30 subscales while decreased age influenced other subscales [33]. Other differences may be due to the PROMs’ comprehensiveness and recall period. The QLQ-C30 summary score covers 15 domains of HRQL across a longer timeframe (1-week) whereas the EQ-5D only captures five domains over the past 1-day and may be more sensitive to daily fluctuations in symptom burden.

### Comparison with other medicinal cannabis studies assessing PROs

Our HRQL findings are consistent with findings from a registry study published in 2021 that found improvements on EQ-5D-5L scores after 1-month in 92 chronic pain patients prescribed MC, which was maintained at 3-months (n = 51) [64]. In contrast, an open-label study comparing MC with usual treatment in 101 neuropathic pain patients, found no improvements in EQ-5D scores after 6-months [24]. While the lack of 3-month data limits direct comparison, it suggests HRQL may return to baseline, reflecting diminishing efficacy, or a response-shift over time [65]. This will be explored further in our 12-month follow-up.

Findings from a double-blind randomized control trial (RCT) published in 2010 investigating 177 cancer pain patients found clinically meaningful and significant improvement in pain intensity Numeric Rating Scores after 2-weeks in the THC:CBD group, but not the THC group, when compared to placebo, however improvements in QLQ-C30 pain scores were not significant [34]. Participants (n = 43) were subsequently followed-up in an open-label study where their QLQ-C30 pain scores improved by 24% over 5-weeks [35]. While our sample was not limited to cancer pain, overall this is consistent with our findings of clinically meaningful improvements in pain scores for chronic pain patients after 2-weeks and maintained over 3-months.

Similar to our findings, significant improvements in fatigue after 3-months of MC therapy have previously been reported in observational studies of patients with multiple sclerosis (n = 389) [66], chronic pain (n = 248) [67], and cancer (n = 743) [68].

Contrary to our findings, an observational study published in 2021 found significant improvements in Pittsburgh Sleep Quality Index scores after 3-months in 36 chronic pain patients prescribed CBD:THC [69]. Although limited by a small sample size, similar improvements were found for 25 insomnia patients with CBD oil over a 1-month period [70]. Another observational study of real-time symptom relief in 409 people with self-reported insomnia found significant improvements in a single-item sleep outcome when inhaling cannabis flower with higher levels of CBD [71], although the authors did not report follow-up time periods, use validated PROMs, or confirm diagnoses. Nevertheless, their findings suggest the way in which MC is administered (inhaled vs ingested) may affect efficacy, and that MC products with a higher ratio of CBD to THC may help promote sleep. Previous research has found that THC and CBD can disrupt the normal sleep cycle by reducing the production of melatonin, a hormone involved in regulating sleep, and that this was dose-dependent [72]. Although, an RCT of 21 chronic pain patients found smoking cannabis containing THC improved sleep outcomes when compared with placebo [23]. Participants with insomnia in our study received a variety of MC products, more than half had formulations containing both CBD and THC (S2 Table), and a large proportion of participants (86%) had more than one health condition. Considering we observed improvements in all other PROs, it is possible that insomnia participants titrated product dosages to gain improvements in another outcome without adequately titrating a product to sufficiently improve sleep, or their sleep was affected by products with high THC levels.

An observational study of MC patients (n = 51) found significant improvements in depression scores after 3-months [73], while another larger study found improvements in depression after 1-month (n = 787) and 3-months (n = 757) in chronic pain patients [74], however neither study reported the clinical meaningfulness. Similar to our findings, clinically meaningful improvements in depression scores were reported for MC patients in a real-world setting with moderate to severe depression (n = 115) after 3-months, but not for MC patients with mild symptoms (n = 157) [75]. The same study reported improvements in anxiety were also limited to patients with high baseline anxiety scores, which was similar to our study findings for patients with anxiety-related conditions.

### Strengths and limitations

Our study assessed a large real-world cohort with a wide range of chronic conditions using validated, condition-relevant PROMs at clinically meaningful time-points and reported the clinical meaningfulness of findings referencing predefined MCIDs. We included everyone within a year time-period, recruited from multiple sites across different Australian states. Product dosing reflected clinical practice use rather than the typically large CBD or THC doses in RCTs [76]. The use of de-identified electronic data collection, with outcomes blinded to doctors and participants throughout the study, reduced the risk of response bias and biases that may be introduced by researchers or clinicians when collecting (or failing to collect) questionnaire data in person.

However, our findings should be interpreted in the context of a single arm study without a control group. A systematic review of cannabis and HRQL studies revealed small effect sizes in RCTs and large effect sizes without control groups [77]. There is a chance that observed improvements are partly due to placebo effect [78], with the widespread public discussion (press and social media) on the benefits of medicinal cannabis and its interaction with the endocannabinoid system increasing patients’ expectations.

Considering MC is a relatively new therapy in Australia, it is likely that patient outcomes are correlated with clinician experience, particularly experience prescribing MC. Unfortunately, we did not collect data related to clinician experience and we were unable to run an analysis clustered by clinician due to the large number of clinicians involved in the study (n = 120) and their uneven participant recruitment numbers (ranging from 1 to 214). For example, two clinicians enrolled more than 200 participants each in the study, whereas 61 clinicians had less than 10 (with 25 clinicians only recruiting one participant). Future research should include clinicians’ MC prescribing experience.

Participants completing follow-up PROMs were likely to be those still using MC because it was helpful to them. The only financial incentive for participants was in accessing discounted MC. Even with the discount, many participants would likely have experienced some financial burden, particularly those who were unemployed or had lower levels of income. This may introduce further bias in the results. Many participants failed to complete PROMs at scheduled follow-up timepoints and did not provide a reason. Some of these participants may have paused or informally withdrawn due to side-effects or lack of MC therapy benefit. Our 12-month follow-up study will identify those who withdrew (rather than missed assessments) and adjust analyses accordingly.

We did not measure adverse events as part of the study. However, follow-up PROMs asked participants to indicate whether they were currently using MC and to select a reason for pausing or withdrawing from the study, which included an option ‘not taking MC due to side-effects.’ Participants and clinicians were also advised to reporting concerning side-effects to the product manufacturer. No participants reported significant adverse effects, or side-effects that they were unable to self-manage through dose-reduction.

We did not include an overall analysis of outcomes by MC product because the participant data we collected did not specify which condition was being treated by each MC product. Many participants (23%) were prescribed more than one MC product, and over half of the total sample (n = 1233) were being treated for multiple conditions. This limitation is also noted in S2 Table which shows participants’ health conditions and prescribed MC products at baseline. Future analyses of outcomes for specific groups will explore this further.

Lastly, our study only observed four MC oil products which further limits the generalization of results to other MC products.

### Clinical implications

Our findings suggest that prescribing MC in clinical practice may alleviate symptoms of pain, fatigue, anxiety, and depression in patients with chronic health conditions and improve overall HRQL. We did not find any evidence of improvements (nor deteriorations) in sleep outcomes for patients prescribed MC in practice. More research is needed to understand the full effects of MC for treating sleep-related conditions, and possibly identify optimal MC formulations, dosing, and routes of administration. Current clinical guidelines recommend clinicians consider trialing MC with patients who have chronic conditions not responding to first line treatments [79], but acknowledge more evidence and education is needed for clinicians to put this into practice. At the very least, prescribing MC may avoid potential risks of cannabis abuse by self-medicating while enabling clinicians to monitor possible adverse effects.

## Conclusion

Short-term findings over 3-months indicate that patients prescribed MC in practice have improved HRQL and reduced fatigue. Patients experiencing anxiety, depression, or chronic pain also improved in those outcomes over 3-months, but no changes in sleep disturbance were observed in patients with sleep disorders. The study continues to follow patients over 12-months to determine whether improvements in PROs are maintained long-term. In addition, further subgroup analyses will be undertaken to determine whether patients with specific health conditions have better outcomes compared with others when using validated condition-specific questionnaires. The findings from this study contribute to the ongoing evidence for decision making both in clinical practice and at policy level.

## Supporting information

S1 Fig Change in categorized severity levels of anxiety and depression between baseline and mean follow-up. (PDF)

S2 Fig Change in Mean EQ-5D-5L Utility Scores and QLQ-C30 Summary Scores over study period stratified by time on study *(with standard error bars)*. (PDF)

S1 Table Location, ethnicity, and gender identity of 2327 participants in the QUEST Initiative study. (PDF)

S2 Table Health Condition Diagnoses of 2327 QUEST participants included in analysis, and medicinal cannabis products prescribed. (PDF)

S3 Table Strobe checklist. (PDF)

## Abbreviations

CBD: Cannabidiol
DASS21: 21 item Short Form for Depression Anxiety and Stress Scale
EORTC: European Organization for Research & Treatment of Cancer
EQ-5D: EuroQol five dimension scale for measuring generic health status
FACIT-Fat: PROMIS Short Form for Fatigue
HRQL: Health-Related Quality of Life
LGP: Little Green Pharma Ltd
MC: Medicinal Cannabis
PROM: Patient-Reported Outcome Measure
PROMIS: Patient-Reported Outcomes Measurement Information System
PRO: Patient Reported Outcomes
QLQ-C15: 15 item version of QLQ-C30 for Palliative Care patients
QLQ-C30: Generic Quality of Life Questionnaire for Cancer patients developed by the EORTC
RCT: Randomized Clinical Trial
SD: Standard Deviation
TGA: Australian Therapeutic Goods Administration
THC: delta-9-tetrahydrocannabinol
US: United States
