# Supplementary material for: Health-related quality of life in patients accessing medicinal cannabis in Australia: The QUEST initiative results of a 3-month follow-up observational study
Source: PLoS One. 2023 Sep 6;18(9):e0290549. doi: 10.1371/journal.pone.0290549 (PMC10482296; doi:10.1371/journal.pone.0290549)

S3 Fig. Change in categorized severity levels of anxiety and depression between baseline and mean follow-up.

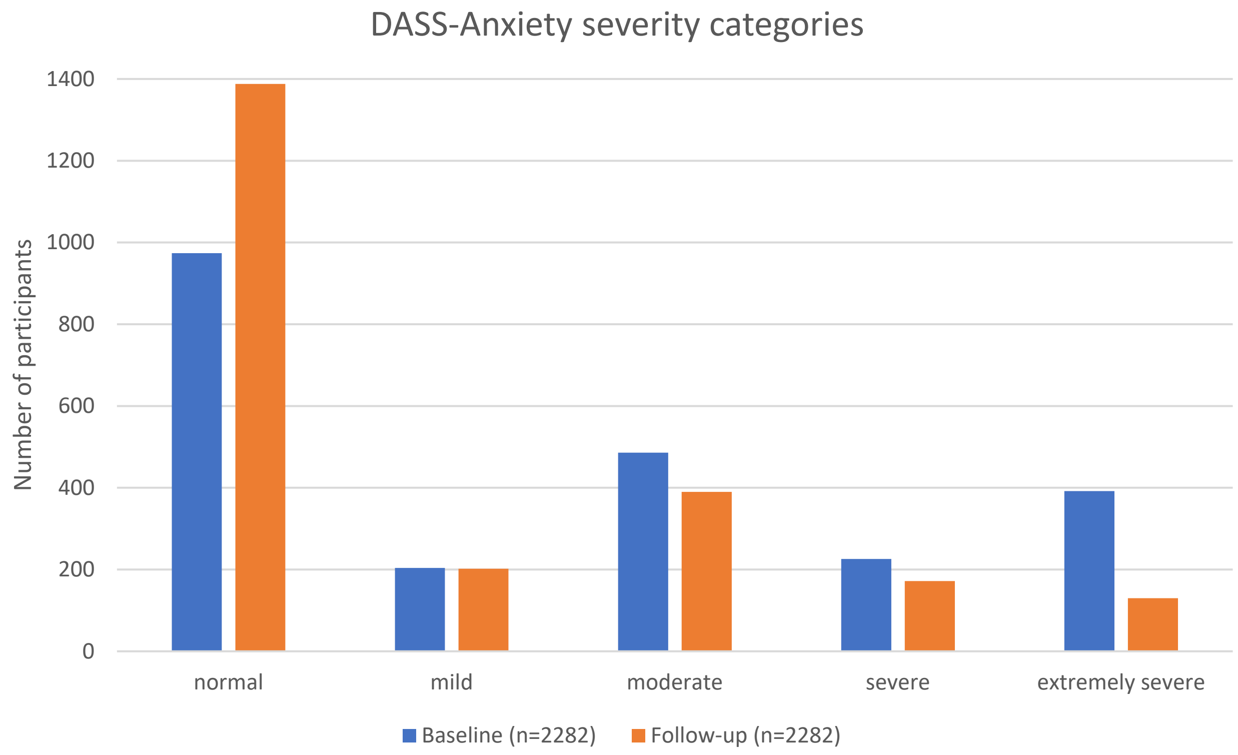

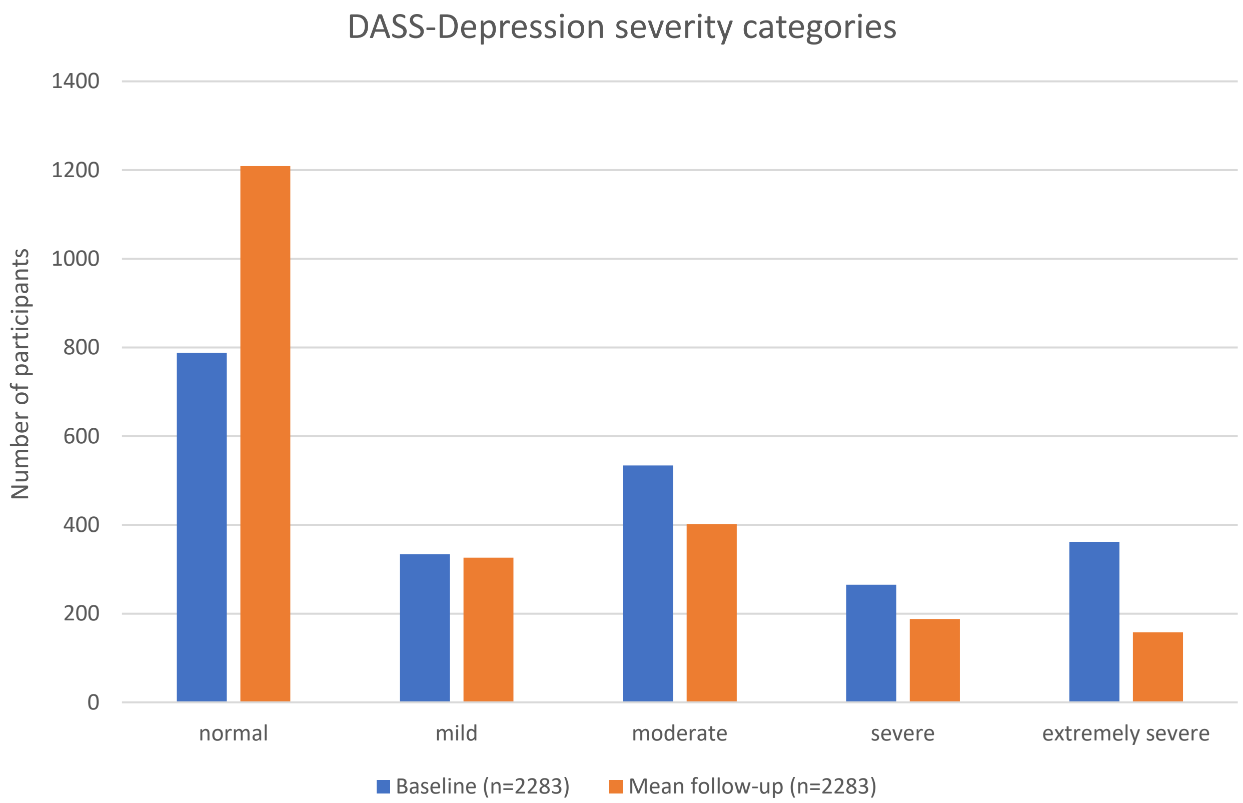

Supplement: S1 Fig — (PDF) [file pone.0290549.s001.pdf]
