# Supplementary material for: Health-related quality of life in patients accessing medicinal cannabis in Australia: The QUEST initiative results of a 3-month follow-up observational study
Source: PLoS One. 2023 Sep 6;18(9):e0290549. doi: 10.1371/journal.pone.0290549 (PMC10482296; doi:10.1371/journal.pone.0290549)

S4 Fig. Change in Mean EQ-5D-5L Utility Scores and QLQ-C30 Summary Scores over study period stratified by time on study *(with standard error bars)*

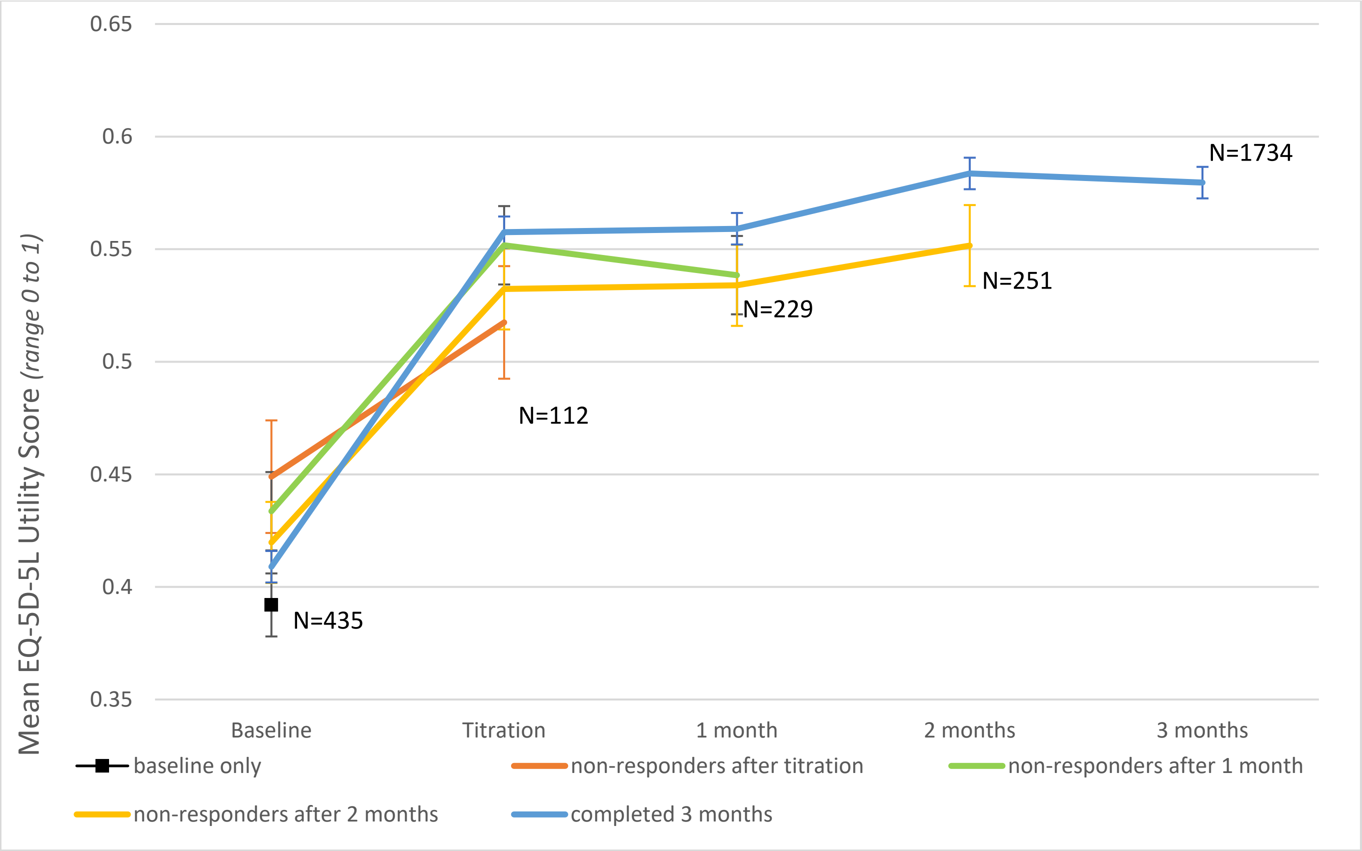

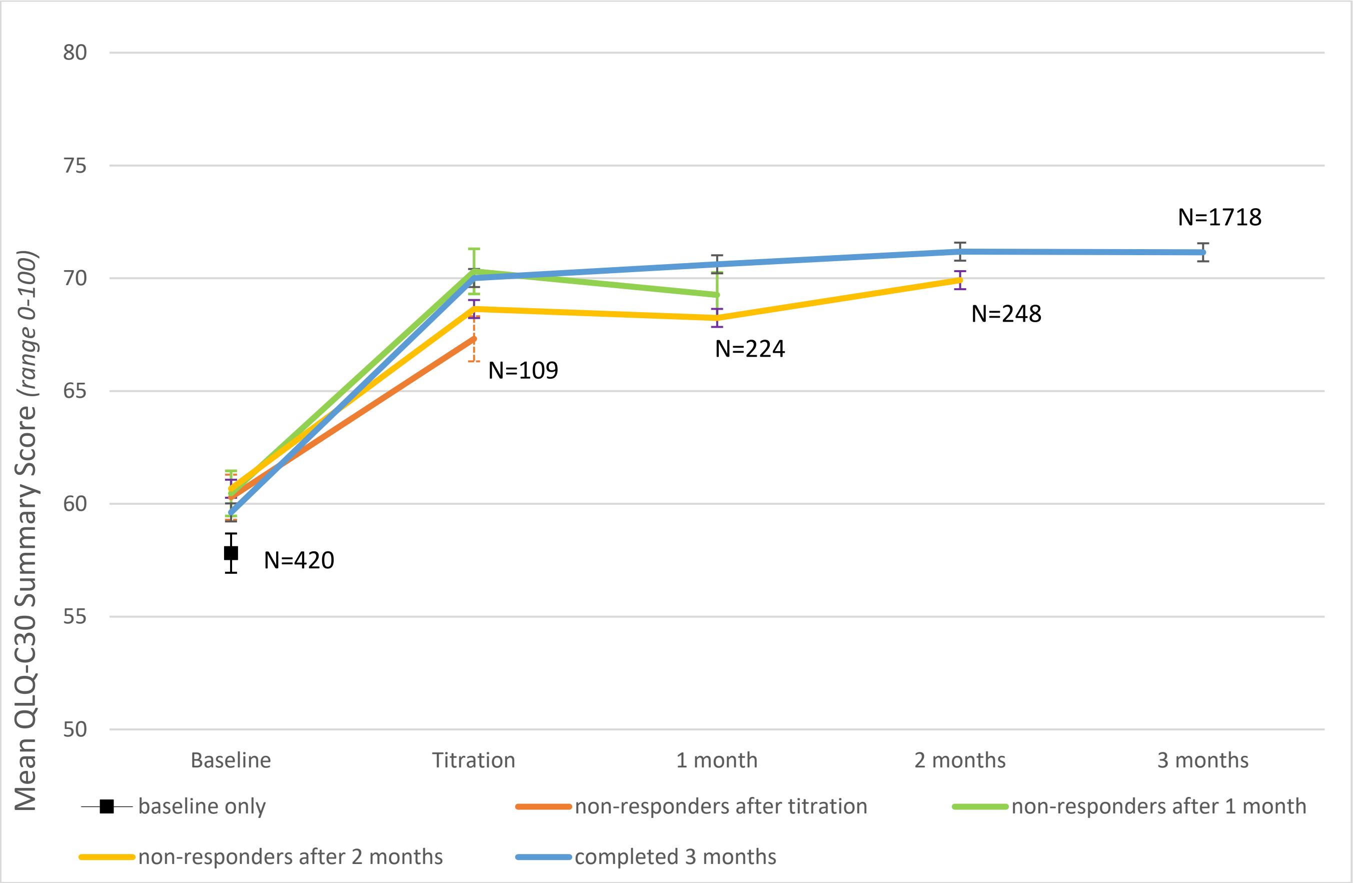

Supplement: S2 Fig — (PDF) [file pone.0290549.s002.pdf]
