# Supplementary material for: Health-related quality of life in patients accessing medicinal cannabis in Australia: The QUEST initiative results of a 3-month follow-up observational study
Source: PLoS One. 2023 Sep 6;18(9):e0290549. doi: 10.1371/journal.pone.0290549 (PMC10482296; doi:10.1371/journal.pone.0290549)
Supplement: S1 Table — (PDF) [file pone.0290549.s003.pdf]

S1 Table. Location, ethnicity, and gender identity of 2327 participants in the QUEST Initiative study.

**Australian Location**	**N=2327**	**%**
New South Wales	899	38.6
Victoria	225	9.7
Queensland	348	15.0
South Australia	103	4.4
Western Australia	743	31.9
Australian Capital Territory	9	0.4
**Ethnicity**[a]		
Aboriginal and/or Torres Strait Islander	54	2.3
Other Australian	1297	55.7
New Zealander	64	2.8
Melanesian and Papuan, Micronesian, or Polynesian	5	0.2
British	371	16.0
Irish	69	3.0
North-West European	117	5.0
Southern and Eastern European	123	5.3
North African and Middle Eastern	18	0.8
South-East Asian	19	0.8
North-East Asian	21	0.9
Southern and Central Asian	23	1.0
North American	35	1.5
Central and South American	30	1.3
Sub-Saharan African	25	1.0
Missing	56	2.4
**Gender Identity**		
Man	813	34.9
Woman	1372	59.0
Transgender	2	0.1
Genderqueer	4	0.2
Agender	2	0.1
Genderless	1	-
Non-binary	13	0.6
Trans man	1	-
Two-spirit	1	-
Genderfluid	4	0.2
Prefer not to disclose	6	0.3
Missing	108	4.6

[a] Self-selected options from list adapted from Australian Bureau of Statistics – Australian Standard Classification of Cultural and Ethnic groups, 2019.
