# Supplementary material for: Health-related quality of life in patients accessing medicinal cannabis in Australia: The QUEST initiative results of a 3-month follow-up observational study
Source: PLoS One. 2023 Sep 6;18(9):e0290549. doi: 10.1371/journal.pone.0290549 (PMC10482296; doi:10.1371/journal.pone.0290549)
Supplement: S2 Table — (PDF) [file pone.0290549.s004.pdf]

S2 Table. Health Condition Diagnoses of 2327 QUEST participants included in analysis, and medicinal cannabis products prescribed.

		LGP medicinal cannabis Products Prescribed[b]										
**Health Condition**	n (%)[a]	1	2	3	4	1,2	1,3	1,4	2,3	2,4	3,4	1,2,3,4
Pain - Chronic neuropathic [MG30.5]	537 (23.1)	182	11	48	204	4	9	44	-	32	2	-
Pain - Chronic widespread [MG30.01]	211 (9.1)	51	3	17	111	1	1	15	-	11	1	-
Pain - Chronic primary visceral [MG30.00]	96 (4.1)	24	1	7	44	-	-	7	-	13	-	-
Pain - Chronic secondary visceral [MG30.4]	18 (0.8)	3	-	3	10	-	-	1	-	1	-	-
Pain - Chronic primary musculoskeletal [MG30.02]	777 (33.4)	173	9	79	388	1	7	64	-	52	3	1
Pain - Chronic secondary musculoskeletal [MG30.3]	107 (4.5)	38	4	20	35	-	-	5	-	2	3	-
Pain - Chronic primary headache/orofacial [MG30.03]	109 (4.6)	22	1	8	60	-	2	9	-	7	-	-
Pain - Chronic secondary headache/orofacial [MG30.6]	18 (0.8)	4	-	1	10	-	-	-	-	2	1	-
Pain - Chronic cancer related [MG30.1]	33 (1.4)	15	-	-	15	-	-	1	1	-	-	-
Pain - Chronic postsurgical or post traumatic [MG30.2]	10 (0.4)	4	1	1	4	-	-	-	-	-	-	-
Movement Disorder - Parkinsonism [8A00]	27 (1.2)	6	-	4	11	-	1	5	-	-	-	-
Movement Disorder - Primary dystonia [8A02.0]	2 (0.1)	-	-	1	1	-	-	-	-	-	-	-
Movement Disorder - Secondary dystonia [8A02.1]	2 (0.1)	-	1	1	-	-	-	-	-	-	-	-
Movement Disorder - Functional dystonia [8A02.3]	3 (0.1)	1	-	1	1	-	-	-	-	-	-	-
Movement Disorder - Paroxysmal dystonia [8A02.2]	9 (0.4)	1	1	3	2	-	1	-	-	1	-	-
Movement Disorder - Ataxia [8A03]	1	-	1	-	-	-	-	-	-	-	-	-
Movement Disorder - Tremor [8A04]	10 (0.4)	2	-	1	5	-	1	1	-	-	-	-
Movement Disorder - Tic disorders [8A05]	1	-	-	-	1	-	-	-	-	-	-	-
Epilepsy or seizures - [8A60]	18 (0.8)	3	1	2	9	-	-	1	-	1	1	-
Nausea and vomiting [MD90]	6 (0.3)	2	-	-	3	-	-	1	-	-	-	-
Inflammatory bowel disease [DD7Z] & IBS	20 (0.9)	3	-	4	13	-	-	-	-	-	-	-
Cachexia [MG20]	6 (0.3)	2	-	1	2	-	1	-	-	-	-	-
Anorexia Nervosa [6B80]	5 (0.2)	1	-	-	3	-	-	-	-	1	-	-
Recurrent depressive disorder [6A71]	25 (1.1)	2	-	1	22	-	-	-	-	-	-	-
Mixed depressive and anxiety disorder [6A73]	256 (11)	50	5	23	139	-	4	19	2	12	1	-
Bipolar disorder [6A60]	11 (0.5)	1	1	2	6	1	-	-	-	-	-	-
Generalised anxiety disorder [6B00]	503 (21.6)	85	9	29	315	3	2	28	1	28	2	1
Post-traumatic stress disorder [6B40]	124 (5.3)	42	5	9	54	1	2	7	-	4	-	-
Chronic insomnia [7A00]	534 (22.9)	153	12	36	243	-	5	34	3	46	1	-
Attention deficit disorder [6A05]	15 (0.6)	6	-	2	4	-	-	1	-	1	-	-
Other	72 (3.1)	19	3	6	33	-	-	1	-	1	2	-

[a] % does not add up to 100 as patients can have up to two health conditions.

[b] Products: 1= LGP Classic 10:10 (10mg THC and 10mg CBD, per mL); 2= LGP Classic 20:5 (20mg THC and 5mg CBD, per mL); 3= LGP Classic 1:20 (1mg THC and 20mg CBD, per mL); 4= LGP Classic CBD 50 (50mg CBD, per mL).

Note: Data on medicinal cannabis products was collected according to participant, not according to health condition. As a result, each condition in the table lists all products participants with that condition received, including products they received for any other condition. For this reason, the table should not be regarded as product indication recommendations.
