# Supplementary material for: Health-related quality of life in patients accessing medicinal cannabis in Australia: The QUEST initiative results of a 3-month follow-up observational study
Source: PLoS One. 2023 Sep 6;18(9):e0290549. doi: 10.1371/journal.pone.0290549 (PMC10482296; doi:10.1371/journal.pone.0290549)
Supplement: S3 Table — (PDF) [file pone.0290549.s005.pdf]

STROBE Statement—checklist of items that should be included in reports of observational *cohort* studies

	Item No.	Recommendation	Page No.	Relevant text from manuscript
**Title and abstract**	1	(*a*) Indicate the study's design with a commonly used term in the title or the abstract	1	3-month follow-up observational study
		(*b*) Provide in the abstract an informative and balanced summary of what was done and what was found	2	Abstract - AMRaC structured
**Introduction**				
Background/rationale	2	Explain the scientific background and rationale for the investigation being reported	4	Introduction - First 3 paragraphs
Objectives	3	State specific objectives, including any prespecified hypotheses	5	This study is reporting the… which is assessing… We hypothesised that…
**Methods**				
Study design	4	Present key elements of study design early in the paper	5	Methods - Study population and design
Setting	5	Describe the setting, locations, and relevant dates, including periods of recruitment, exposure, follow-up, and data collection	5-6	Methods - Study population and design<br>Methods - Data Collection
Participants	6	(*a*) *Cohort study*—Give the eligibility criteria, and the sources and methods of selection of participants. Describe methods of follow-up	5-6	Methods - Study population and design<br>Methods - Data Collection
		(*b*) *Cohort study*—For matched studies, give matching criteria and number of exposed and unexposed	n/a	
Variables	7	Clearly define all outcomes, exposures, predictors, potential confounders, and effect modifiers. Give diagnostic criteria, if applicable	5<br><br>7-8	Methods - Study population and design<br>Methods - Data Collection Table 1
Data sources/ measurement	8	For each variable of interest, give sources of data and details of methods of assessment (measurement). Describe comparability of assessment methods if there is more than one group	7-8	Methods - Data Collection Table 1
Bias	9	Describe any efforts to address potential sources of bias	20-21	Discussion - Strengths and limitations

	Item No.	Recommendation	Page No.	Relevant text from manuscript
Study size	10	Explain how the study size was arrived at	9	Methods – Statistical significance
Quantitative variables	11	Explain how quantitative variables were handled in the analyses. If applicable, describe which groupings were chosen and why	7-9	Methods – Data Collection Table 1<br>Methods – Statistical significance
Statistical methods	12	(*a*) Describe all statistical methods, including those used to control for confounding	9<br>13	Methods – Statistical significance<br>Results - PROs
		(*b*) Describe any methods used to examine subgroups and interactions	9-10	Methods – Statistical analysis
		(*c*) Explain how missing data were addressed	9	Methods – Statistical significance
		(*d*) *Cohort study*—If applicable, explain how loss to follow-up was addressed	9<br>17	Methods - Study population and design<br>Results – Missed assessments
		(*e*) Describe any sensitivity analyses	9-10	Methods – Statistical analysis
**Results**				
Participants	13	(a) Report numbers of individuals at each stage of study—eg numbers potentially eligible, examined for eligibility, confirmed eligible, included in the study, completing follow-up, and analysed	10	Results<br>Figures 1-3<br>Table 3
		(b) Give reasons for non-participation at each stage		Results Figure 1
		(c) Consider use of a flow diagram		Results Figure 1
Descriptive data	14	(a) Give characteristics of study participants (eg demographic, clinical, social) and information on exposures and potential confounders	11	Results<br>and Table 2; Appendix A; Appendix B
		(b) Indicate number of participants with missing data for each variable of interest		Figures 2-3
		(c) *Cohort study*—Summarise follow-up time (eg, average and total amount)	6	Methods – Data Collection
Outcome data	15	*Cohort study*—Report numbers of outcome events or summary measures over time	13-17	Results<br>and Table 3

	Item No.	Recommendation	Page No.	Relevant text from manuscript
Main results	16	(*a*) Give unadjusted estimates and, if applicable, confounder-adjusted estimates and their precision (eg, 95% confidence interval). Make clear which confounders were adjusted for and why they were included	13-17	Results and Table 3
		(*b*) Report category boundaries when continuous variables were categorized	8	*Methods - Table 1*<br>*Results - Figure 3*<br>*Appendix c*
		(*c*) If relevant, consider translating estimates of relative risk into absolute risk for a meaningful time period	n/a	
Other analyses	17	Report other analyses done—eg analyses of subgroups and interactions, and sensitivity analyses	13-17	Results
**Discussion**				
Key results	18	Summarise key results with reference to study objectives	18	Discussion – Principal findings
Limitations	19	Discuss limitations of the study, taking into account sources of potential bias or imprecision. Discuss both direction and magnitude of any potential bias	20-21	Discussion – Strengths and limitations
Interpretation	20	Give a cautious overall interpretation of results considering objectives, limitations, multiplicity of analyses, results from similar studies, and other relevant evidence	22	Discussion – Clinical implications
Generalisability	21	Discuss the generalisability (external validity) of the study results	20-21	Discussion – Strengths and limitations
**Other information**				
Funding	22	Give the source of funding and the role of the funders for the present study and, if applicable, for the original study on which the present article is based	24	Declarations - Funding

Note: An Explanation and Elaboration article discusses each checklist item and gives methodological background and published examples of transparent reporting. The STROBE checklist is best used in conjunction with this article (freely available on the Web sites of PLoS Medicine at http://www.plosmedicine.org/, Annals of Internal Medicine at http://www.annals.org/, and Epidemiology at http://www.epidem.com/). Information on the STROBE Initiative is available at www.strobe-statement.org.
